# Degradation of toluene by ortho cleavage enzymes in *Burkholderia fungorum* FLU100

**DOI:** 10.1111/1751-7915.12147

**Published:** 2014-08-18

**Authors:** Daniel Dobslaw, Karl-Heinrich Engesser

**Affiliations:** Department of Biological Waste Air Purification, Institute of Sanitary Engineering, Water Quality and Solid Waste Management, University of StuttgartBandtäle 2, Stuttgart, D-70569, Germany

## Abstract

*Burkholderia fungorum* FLU100 simultaneously oxidized any mixture of toluene, benzene and mono-halogen benzenes to (3-substituted) catechols with a selectivity of nearly 100%. Further metabolism occurred via enzymes of *ortho* cleavage pathways with complete mineralization. During the transformation of 3-methylcatechol, 4-carboxymethyl-2-methylbut-2-en-4-olide (2-methyl-2-enelactone, 2-ML) accumulated transiently, being further mineralized only after a lag phase of 2 h in case of cells pre-grown on benzene or mono-halogen benzenes. No lag phase, however, occurred after growth on toluene. Cultures inhibited by chloramphenicol after growth on benzene or mono-halogen benzenes were unable to metabolize 2-ML supplied externally, even after prolonged incubation. A control culture grown with toluene did not show any lag phase and used 2-ML as a substrate. This means that 2-ML is an intermediate of toluene degradation and converted by specific enzymes. The conversion of 4-methylcatechol as a very minor by-product of toluene degradation in strain FLU100 resulted in the accumulation of 4-carboxymethyl-4-methylbut-2-en-4-olide (4-methyl-2-enelactone, 4-ML) as a dead-end product, excluding its nature as a possible intermediate. Thus, 3-methylcyclohexa-3,5-diene-1,2-diol, 3-methylcatechol, 2-methyl muconate and 2-ML were identified as central intermediates of productive *ortho* cleavage pathways for toluene metabolism in *B. fungorum* FLU100.

## Introduction

Because of widespread use of alkyl as well as halogen benzenes as reactants and solvents in chemical industry, they belong to the top ten of most frequently appearing contaminants in water and soil (UBA, 2013). Biological decontamination of these sites is highly interesting because of its low costs and ‘low-tech’ character (Christodoulatos *et al*., 1996; Johnson and Odencrantz, 1999; Kao and Borden, 1999; SMUL Sachsen, 2000; Kao and Prosser, 2001; Kao *et al*., 2006; Farhadian *et al*., 2008). In contrast, degradation of cocktails of contaminants is still problematic (Corseuil *et al*., 1998; Lovanh *et al*., 2002), particularly with regard to mixtures of chloro- and methyl-substituted aromatics.

Chlorobenzene is the most widely used mono-halogen benzene. It is firstly converted to (chloro-)catechols as central intermediates via dioxygenation of the aromatic ring forming chlorocyclohexa-3,5-diene-1,2-diols (Reineke and Knackmuss, 1984; Beil *et al*., 1997), via two successive monooxygenations producing chlorophenols as intermediates (Yen *et al*., 1991), or via initial dehalogenation forming phenol (Zhang *et al*., 2011).

Chlorocatechols are usually further transformed by intradiol (*ortho*) ring cleavage forming chloro-*cis/cis*-muconates (Dorn and Knackmuss, 1978; Schlömann, 1994; Reineke, 1998; Zerlin, 2004; Gröning *et al*., 2012). Subsequent conversion is performed by a chloromuconate cycloisomerase directly forming dienelactone (4-carboxymethylenebut-2-en-4-olides) with intermediary dehalogenation (Schmidt *et al*., 1980; Kuhm *et al*., 1990; Vollmer *et al*., 1994; Vollmer and Schlömann, 1995; Reineke, 1998) or indirectly via chloromuconolactones and subsequent dehalogenation (Vollmer *et al*., 1994; Moiseeva *et al*., 2002; Skiba *et al*., 2002; Nikodem *et al*., 2003; Gröning *et al*., 2012). The dienelactones are further cleaved hydrolytically to maleyl acetate being reduced in the next step to 3-oxoadipate, a central metabolite of the 3-oxoadipate pathway (Reineke, 1998). The degradation of fluorobenzene, bromobenzene and iodobenzene proceeds in a way similar to the degradation of chlorobenzene (Strunk, 2000; 2007; Carvalho *et al*., 2006; 2007; 2009; Strunk *et al*., 2006; Moreira *et al*., 2013; Strunk and Engesser, 2013).

Toluene as a widely used alkyl-substituted aromatic compound is either converted to methylcatechols, catechol or 3,4-dihydroxybenzoate as central intermediates. Formation of methylcatechols – similar to chlorocatechols – takes place either via dioxygenation of the aromatic ring forming methylcyclohexa-3,5-diene-1,2-diole (Gibson *et al*., 1974; Zylstra *et al*., 1988; Warhurst *et al*., 1994; Cho *et al*., 2000; Kim *et al*., 2002; Cafaro *et al*., 2005; Chaikovskaya *et al*., 2008) or via two successive monooxygenations with cresols as intermediates. Second, toluene can be transformed to catechol via side-chain oxidation forming benzyl alcohol, benzaldehyde and benzoate as intermediates (Worsey and Williams, 1975; Harayama, 1994). Finally, transformation of toluene to 3,4-dihydroxybenzoate via p-cresol and 4-hydroxybenzoate was also described (Richardson and Gibson, 1984; Shields *et al*., 1989; Kaphammer *et al*., 1990; Yen *et al*., 1991). Subsequently, methylcatechols are usually mineralized by extradiol (*meta*) ring cleavage (Hou *et al*., 1977; Taeger *et al*., 1988; Reineke, 1998).

Whereas the mineralization of alkylated or halogenated aromatics as sole substrates is non-critical with most bacteria, mixtures of those compounds are hardly biodegradable, based preferentially on the incompatibility of individual pathways with potential formation of reactive intermediates in case of the *meta* cleavage pathway (Klecka and Gibson, 1981; Knackmuss, 1981; Bartels *et al*., 1984; Rojo *et al*., 1987; Pettigrew *et al*., 1991). Only a few strains are able to deal with chloro-substituted catechols via *meta* cleavage pathway. Nonetheless, in most of these strains, inactivation of the *meta* pyrocatechase is only partially compensated by reactivation or pricey continuing de novo synthesis (Oldenhuis *et al*., 1989; Haigler *et al*., 1992; Arensdorf and Focht, 1994; 1995; Hollender *et al*., 1994; 1997; Wieser *et al*., 1994; Heiss *et al*., 1997; Mars *et al*., 1997; 1999; Franck-Mokross and Schmidt, 1998; Kaschabek *et al*., 1998; Riegert *et al*., 1998; 1999; Göbel *et al*., 2004).

The second alternative to mineralize mixtures of alkyl- and halogen-substituted aromatics is to degrade not only the halogenated catechols, but also alkyl-substituted aromatics like toluene via *ortho* pathways. The *ortho* cleavage of methylcatechols as an individual reaction was described manifold in literature. For instance, 4-methylcatechol was converted to 4-carboxymethyl-4-methylbut-2-en-4-olide (4-methyl-2-enelactone, 4-methylmuconolactone, 4-ML) being a dead-end metabolite in most strains possessing *ortho* cleavage pathways (Catelani *et al*., 1971; Rojo *et al*., 1987; Sovorova *et al*., 2006; Marín *et al*., 2010). However, *Cupriavidus necator* JMP134 (Pieper *et al*., 1985; 1988; 1990; Bruce *et al*., 1989), *Rhodococcus opacus* 1CP (Sovorova *et al*., 2006) and *Pseudomonas knackmussii* B13 FR1(pFRC20p), a strain engineered genetically (Rojo *et al*., 1987), were able to circumvent this bottle neck by an isomerase enzyme, shifting the methyl group from 4-position to the 3-position in order to get 3-methylmuconolacton (4-carboxymethyl-3-methylbut-2-en-4-olide), which was a growth substrate for each strain.

However, the major obstacle for degradation of toluene via *ortho* cleavage reaction is the fact that mainly 3-methylcatechol is formed as an intermediate of the oxidation of the ring. This intermediate is further transformed to 2-methyl-*cis,cis*-muconic acid followed by formation of 4-carboxymethyl-2-methylbut-2-en-4-olide (2-methyl-2-enelactone, 2-methylmuconolactone, 2-ML), which has frequently been described to be a dead-end metabolite.

So far, only a few authors described a productive mineralization of 2-ML. Taeger *et al*. used 2-ML as a substrate for bacterial enrichments obtaining strains totally mineralizing 2-ML. However, exposing these strains to 3-methylcatechol, enzymes of the *meta* cleavage pathway were induced (Taeger *et al*., 1988). Pettigrew *et al*. found a mutant of *Pseudomonas* sp. JS150, called JS6, which had a defect in the catechol-2,3-dioxygenase and thus was unable to cleave 3-methylcatechol by *meta* pathway (Pettigrew *et al*., 1991). Furthermore, 3-methylcatechol was mineralized by a modified *ortho* pathway with 2-ML as intermediate. However, toluene was no inducer for this *ortho* pathway and the native strain preferred mineralization of toluene by *meta* cleavage pathway. Similar results were found with *Ralstonia* sp. PS12 (Lehning, 1998). Franck-Mokross and Schmidt reported about the strain *Pseudomonas* sp. D7-4 being able to degrade m-toluate productively via 3-methylcatechol and 2-ML by using modified *ortho* enzymatics (Franck-Mokross and Schmidt, 1998). However, 2-ML accumulated temporarily and was further mineralized after a lag phase lasting a few hours. Consistently, this strain was not able to mineralize toluene.

We previously described the strain *Burkholderia fungorum* FLU100 being able to mineralize all mono-halogenated benzenes, benzene and toluene as pure substances by an *ortho* cleavage pathway (Strunk, 2000; 2007; Dobslaw, 2003; Strunk *et al*., 2006; Strunk and Engesser, 2013). We postulated 2-ML as intermediate of the toluene degradation pathway.

In the present paper, we show data clearly demonstrating 2-ML as the principal intermediate in the productive degradation of toluene by modified *ortho* cleavage enzymes as well as the capability of strain FLU100 to degrade mixtures of benzene, toluene and mono-halogen benzenes simultaneously. To our knowledge, this is the first description of a functional, non-engineered *ortho* pathway for total degradation of toluene.

## Results and discussion

### Simultaneous degradation of mixtures of aromatic compounds

*Burkholderia fungorum* FLU100 was able to degrade any mixture of the aromatic compounds benzene, toluene, fluorobenzene, chlorobenzene, bromobenzene as well as iodobenzene simultaneously presented as sole carbon source without observing a lag phase. Increasing the number of compounds, the substrate-specific transformation rates declined. However, the sum of the specific transformation rates stayed nearly constant during each test series. Thus, transformation of each substrate seems to be accomplished by the same initial dioxygenase previously described (Strunk and Engesser, 2013).

Extracts of cultivation media of FLU100 with 0.25% dimethylformamide (DMFA) and 2 to 3 mmol l^−1^ aromatic compounds as start concentrations showed transformation rates between 90 and 120 mg C l^−1^ h^−1^ OD^−1^, and 180 mg C l^−1^ h^−1^ OD^−1^ at maximum in case of optimal induction conditions (Supporting Information Table S1).

Aqueous samples without DMFA, analysed by a high performance liquid chromatography – system with UV/visible light detector (HPLC-UV/VIS), revealed rates up to 280 mg C l^−1^ h^−1^ OD^−1^ in case of toluene as sole carbon source showing the toxic effect of DMFA. However, the substrate-specific transformation rates reflected differences in the affinity of the enzyme for different substrates (in mg C l^−1^ h^−1^ OD^−1^: toluene, 280; benzene, 178; fluorobenzene, 127; chlorobenzene, 159).

As shown, the maximum specific transformation rate for toluene with FLU100 in case of variable concentrations of toluene was 280 mg C l^−1^ h^−1^ OD^−1^ and remained stable with declining toluene concentrations down to 60 mg toluene·l^−1^. Thereby, a K_s_ value of 30 mg toluene⋅l^−1^ could be calculated.

### Influence of pH value on mineralization behaviour of toluene

Transforming high amounts of toluene, benzene or mono-halogen benzenes as sole substrates as well as in any mixture, supernatants and growing cells of FLU100 stained black. This is due to a well known pH-dependent polymerization of (substituted) catechols as soon as they are being accumulated. Hence, the rate of cleavage of catechols is a bottleneck in the degradation pathway for these aromatics impeding up-scaling for full-scale applications. The amount of polymerized products analysed photometrically was reduced by lowering the pH value of the cultivation medium from 7.15 to 5.0. Accordingly, corresponding HPLC analyses showed an increase in the concentration of 2-methylmuconate (Supporting Information Fig. S1) as well as a still unknown metabolite with higher lipophilicity than 3-methylcatechol. Obviously, under this regime, higher amounts of methylcatechol could be enzymatically metabolized without suffering chemical autoxidation.

### Substrate-specific oxygen uptake relationships

Strain FLU100 degrades a wide spectrum of aromatics, favouring a broad application in possible bioremediation. The oxidation potential of the initial enzymes was characterized by measuring the specific oxygen uptake rates for different carbon sources in dependence of fluorobenzene, toluene and benzene as pre-cultivation substrates, allowing to judge the number of initial enzymes by analysis of the relative maximum transformation rates. The corresponding results are shown in Supporting Information Tables S2 and S3. All results are standardized for toluene or catechol as 100% respectively. These data are giving the average result of several independent experiments each performed in double.

With the exception of the value for fluorobenzene in fluorobenzene-grown cells, the relative specific activities for each substrate were nearly independent of the pre-cultivation conditions. Thus, the same initial oxygenase seems to be induced independently from the nature of different benzene derivatives. As only low activities could be measured with the phenol derivatives tested as well as with benzylic alcohol and benzoate, the initial enzyme most probably is a dioxygenase directly oxidizing the aromatic ring. No activity for side chain oxidation, which is a possible reaction in case of toluene, was observed. Currently, we have no proof to explain higher activity for fluorobenzene after growth on fluorobenzene. The existence of a special transport system for fluorobenzene into the cells induced by fluorobenzene is the most probable explanation.

In contrast to the initial dioxygenase, oxygen uptake values of catechol cleavage enzymes strongly depend on the type of growth substrate. The absolute oxygen uptake of nearly 2100 U in case of cells pre-grown on fluorobenzene was 10-fold higher than that measured for toluene cells and 20-fold higher than that of cells pre-grown on benzene (see Supporting Information Table S3).

This intensified expression of catechol cleavage enzymes in case of fluorobenzene may reflect and compensate the lower relative activities for ring cleavage of 3-fluorocatechol in order to avoid its accumulation and autoxidation.

In general, muconates are described as inducers of catechol *ortho* cleavage pathways (Feist and Hegeman, 1969). According to literature, 2-halomuconates are stronger inducers for the modified chlorocatechol pathway than 2-methylmuconate or unsubstituted muconate (Reineke, 1998). However, comparison of the relative activities for muconates in cells pre-grown on fluorobenzene or toluene, respectively, showed high similarity. In contrast, cells grown on benzene were found to strongly deviate from this pattern, indicating the presence of an additional *ortho* pathway, being too specific to tolerate bulky substituents in the substrates. Thus, toluene is mainly transformed by the modified *ortho* pathway with chlorocatechol-1,2-dioxygenase as the initial enzyme, followed by a broad-specificity enzyme like chloromuconate cycloisomerase.

### Proof of dioxygenase nature of the initial enzyme by accumulation of its products

The existence of an initial dioxygenase enzyme was also proofed by a transposon mutant of strain FLU100, called FLU100 P2R5 (Strunk, 2007), where the transposon element was inserted within the benzene dihydrodiol-dehydrogenase encoding gene sequence. During turnover of toluene, the corresponding 3-methylcyclohexa-3,5-diene-1,2-diol accumulated in the supernatant. This diendiol can be re-aromatized also chemically by heat treatment for 5 min under acidic conditions yielding the corresponding o-cresol quantitatively. For all mono-halogen benzenes, toluene and benzene, the corresponding diendiols were transformed to phenolic compounds, which were identified and verified by commercial standards.

Second, the same diendiol structures were produced by *Escherichia coli* pST04, an analogue to strain pSTE44, which contained a lac operon-regulated tetrachlorobenzene dioxygenase. This strain as well as the products of transformation was formerly described in literature in more detail (Pollmann *et al*., 2003). This clearly establishes the nature of 3-methylcyclohexa-3,5-diene-1,2-diol to be the principal intermediate of the oxidation of toluene in FLU100 after growth with toluene.

Furthermore, using FLU100 P2R5, we were able to produce and isolate these diendiol structures for all six aromatic substrates (for diendiol structures of benzene, toluene and fluorobenzene, see Supporting Information Fig. S2) and additionally used them as substrates in conversion experiments. The process of conversion was followed by HPLC analyses. During conversion of the 3-methylcyclohexa-3,5-diene-1,2-diol, for example, a temporary accumulation of further intermediates of toluene degradation, namely 3-methylcatechol, 2-methyl-*cis,cis*-muconic acid, as well as 2-ML was observed, again proposing the way of toluene metabolism to follow the route for halo-benzene degradation up to the level of methyl lactone.

**Fig 1:**
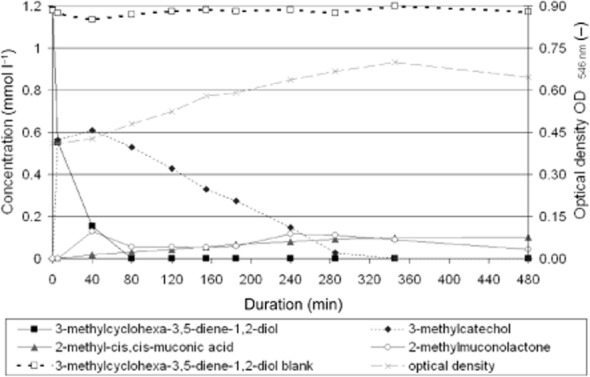
Concentration of 3-methylcyclohexa-3,5-diene-1,2-diol as substrate and its intermediates during conversion by strain FLU100 pre-grown on toluene. Additionally, values of optical density measured at 546 nm wavelength are given.

The conversion rate of the diendiol compound in comparison with conversion rates of 3-methylcatechol, 2-methylmuconic acid and 2-ML was extremely high. In case of 3-methylcyclohexa-3,5-diene-1,2-diol, nearly 0.6 mmol l^−1^ of the substrate, equivalent to 50% of the concentration supplied, was biologically converted to the corresponding catechol within 5 min (Fig. 1). Because of time delays of HPLC analyses, the conversion rate of diendiol between the second and third measurements was transferred to Table 1, showing absolute conversion rates for all relevant substrates and intermediates for cells pre-grown on toluene.

**Table 1:** Maximum substrate specific conversion rates of cultures of FLU100 pre-grown on toluene (column, concentration > 1 mmol l^−1^) in mmol l^−1^ h^−1^ OD^−1^

Intermediate	Substrate				
	Toluene	3-methyldiendiol	3-methylcatechol	2-methylmuconolactone	Succinate
Toluene	3.323				
3-methyldiendiol		1.636			
3-methylcatechol		0.251	0.306		
2-methylmuconic acid		0.219	0.236		
2-methylmuconolactone		0.195	0.219	0.796	
Succinate					3.332

Conversion of produced intermediates was measured after total conversion of the substrate (line, concentration <1 mmol l^−1^). In case of toluene as substrate, no intermediate conversion rates were detected, because the solvent used was not adequate for intermediate measurement.

### Identification of 3-methylcatechol and 2-methyl-cis/cis-muconic acid as further intermediates of the toluene degradation pathway

Corresponding catechols were formed out of the diendiol intermediates by dehydrogenation using viable cells (Fig. 1). Identification was performed using a gas chromatograph with a mass spectrometer as detector (GC-MS) and analysing the concentrated organic phase after a threefold extraction with ethyl acetate as well as methylation of the organic phase with diazomethane. For toluene, 3-methylcatechol (before methylation) and 2-methoxy-3-methylphenol (after methylation) were identified by using commercially available standard compounds (Supporting Information Table S4).

The catechols of all six substrates were cleaved by an *ortho* cleavage pathway and no *meta* cleavage activity was observed. Measurement of absolute and relative conversion activities of whole cells as well as crude extracts of strain FLU100 exhibited an activity pattern with low similarity to the conversion pattern in *P. knackmussii* B13 (Dorn and Knackmuss, 1978). In contrast, the activity pattern of FLU100 revealed high similarity to the activity pattern typical for chlorocatechol-1,2-dioxygenase of B13 readopted by the authors (Dobslaw, 2003).

Even though strain FLU100 is able to metabolize 4-substituted catechols, the main, nearly exclusively formed transformation products of the benzene derivatives examined in this study are the corresponding 3-substituted catechols. These catechols are further cleaved to the corresponding (2-substituted) *cis-cis*-muconic acids. These muconic acids were identified by comparison of retention times and wavelength spectra with reference substances produced by *E. coli* Klon 4, carrying plasmid pBBRCI, from the corresponding catechols. This mutant expressed an chlorocatechol-1,2-dioxygenase regulated by a lac operon and was previously described (Perez-Pantoja *et al*., 2003).

### The intermediate nature of 2-methylmuconolactone in the toluene degradation pathway of FLU100

While 2-halogenated muconic acids can be transformed to *trans*-dienlactone during lactone ring formation/elimination of halide by muconate-cycloisomerases (see for example Vollmer *et al*., 1994; Vollmer and Schlömann, 1995; Reineke, 1998; Moiseeva *et al*., 2002), 2-methylmuconic acid as intermediate of toluene cannot be transformed this way, because the methyl group is no leaving group at all. We observed that the cycloisomerase transformed 2-methylmuconic acid to 2-methylmuconolactone (2-ML), which was further mineralized. There was no indication for the existence of an exocyclic 5-methylmuconolactone, a possible product of alternative cycloisomerization of 2-methylmuconic acid.

In contrast to strain *Pseudomonas sp.* D7-4 (Franck-Mokross and Schmidt, 1998; Taeger *et al*., 1988;) and other strains obtained by enrichment on 2-ML and 3-methylbenzoate (Taeger *et al*., 1988), which were also able to mineralize 2-ML, strain FLU100 was able to grow on toluene as sole source of carbon and energy.

**Fig 2:**
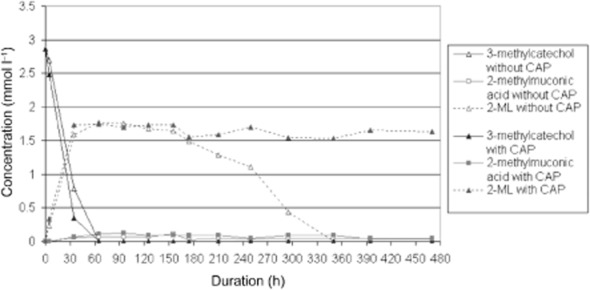
Concentration of 3-methylcatechol as substrate and its transformation products during conversion by strain FLU100 pre-grown on benzene (OD_546nm_ = 1.5; double tested). One row contained chloramphenicol (CAP) as cytostatic drug avoiding induction of new enzymes. 2-methylmuconolactone (2-ML) was only transformed in absence of CAP.

**Fig 3:**
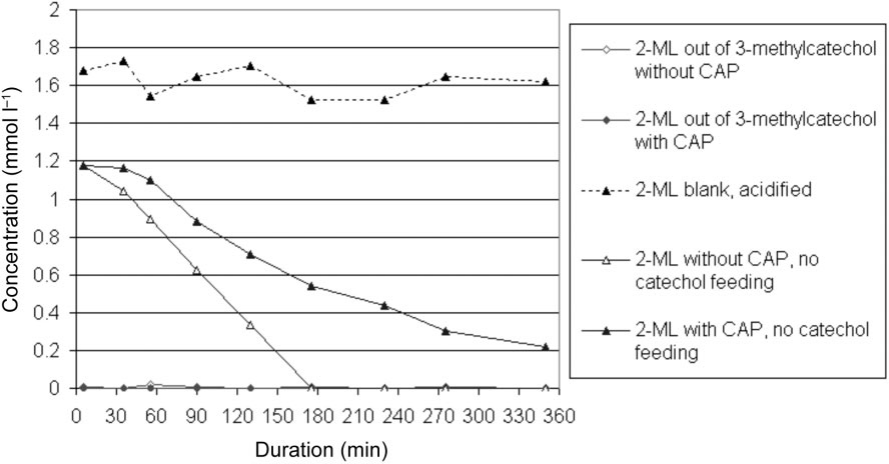
Concentration of 2-methylmuconolactone (2-ML) as intermediate of the conversion of 3-methylcatechol as well as sole substrate by whole cells of strain FLU100 pre-grown on toluene. To the second flask of each test row, chloramphenicol (CAP) as cytostatic drug was added to avoid induction of new enzymes. The optical density was OD_546nm_ = 1.6. In all cases, 2-ML was transformed showing induction of relevant enzymes. Additionally, the concentration of 2-ML in a cell-free supernatant at a pH value of 1–2 as a blank row is presented.

However, transforming 3-methylcatechol by cells of strain FLU100 pre-grown on benzene or mono-halogen benzenes, nearly 55–65% of the provided 3-methylcatechol temporarily accumulated as 2-ML. Further transformation was observed after a lag phase of 2–3 h (Fig. 2). By contrast, no or only small concentrations of 2-ML accumulated in case of toluene-induced/growing cells (Fig. 3). This means that key enzymes for degradation of 2-ML were only induced after adaptation on toluene, but not on benzene or mono-halogen benzenes.

To prove this interpretation and to avoid induction of new enzymes, the same experiments were done after addition of chloramphenicol (CAP) as an inhibitor of protein synthesis. Cells induced with benzene or mono-halogen benzenes showed similar concentrations of 2-ML accumulating in the supernatant compared with non-inhibited cells. However, no further transformation appeared, even after prolonged incubation (Fig. 2). In contrast, no accumulation of 2-ML was detected when cells used were previously induced with toluene (Fig. 3).

In a second experiment, an aqueous solution of 2-ML was produced by complete transformation of 3-methycatechol by cells of FLU100 grown on benzene, followed by subsequent separation of the cells. The final concentration of 2-ML was adjusted to about 1.7 mmol l^−1^. This solution was split and added to two liquid cultures of strain FLU100 pre-grown on toluene. One of these cultures has been supplemented with CAP. Both cultures transformed 2-ML showing no lag phase at transformation rates of 0.35 mmol l^−1^ h^−1^ OD^−1^ (culture without CAP) and 0.17 mmol l^–1^ h^–1^ OD^−1^ (with CAP) respectively. The transformation rate without CAP was twofold higher, because the formation of new enzymes was possible (Fig. 3). The results of both experimental concepts confirmed the induction of 2-ML-specific transforming enzymes in case of toluene-metabolizing cells of FLU100.

2-ML as metabolite was identified by HPLC-MS and GC-MS analyses and identity was proofed by comparison with data of Knackmuss and colleagues (1976) and Pieper and colleagues (1985), given in Table 2.

**Table 2:** Comparison of detected GC-MS fragments and corresponding intensities of the identified metabolites 2-methylmuconolactone (2-ML, column 2) and 4-methymuconolactone (4-ML, column 5) of strain FLU100 with data of 2-methylmuconolactone (column 3), 3-methylmuconolactone (3-ML, column 4) and 4-methylmuconolactone (column 6) given by Knackmuss and colleagues (1976) and Pieper and colleagues (1985)

m/z	Intermediate 2-ML of strain FLU100; intensity (%)	Knackmuss *et al*., 1976 2-ML; intensity (%)	Pieper *et al*., 1985 3-ML; intensity (%)	Intermediate 4-ML of strain FLU100; intensity (%)	Knackmuss *et al*., 1976 4-ML; intensity (%)
156	1.5	3.0	7.9	8.8	17.0
141	0.0	0.0	n.d.	8.4	13.0
139	1.4	>0.0	n.d.	n.d.	n.d.
138	n.d.	n.d.	12.4	n.d.	n.d.
128	n.d.	n.d.	n.d.	3.6	n.d.
127	1.7	n.d.	n.d.	n.d.	n.d.
111	n.d.	n.d.	11.6	15.2	n.d.
110	100.0	100.0	82.8	15.2	22.0
99	0.8	n.d.	n.d.	36.0	n.d.
97	31.6	30.0	51.1	100.0	100.0
96	n.d.	n.d.	22.4	n.d.	n.d.
69	48.8	n.d.	100.0	41.6	n.d.
68	n.d.	n.d.	37.5	n.d.	n.d.
43	4.0	n.d.	n.d.	36.0	n.d.
41	34.4	n.d.	n.d.	n.d.	n.d.
					
λ_max_	208 nm	210 nm	n.d.	208 nm	210 nm

The fragment with the highest intensity is normalized to 100% and other fragments are given as relative intensities. Additionally, the wave length at maximum absorption is given in the last line.

n.d. = not detected or intensity not given in literature.

A theoretical alternative dioxygenation route for toluene in 3,4-position, i.e. at *meta* and *para* position forming 4-methylcyclohexa-3,5-diene-1,2-diol, 4-methylcatechol, 3-methyl-*cis,cis*-muconic acid and 4-ML as intermediates, was found to be non-productive. Metabolism of 4-methylcatechol, added to whole cells of FLU100, lead to stoichiometric accumulation of 4-ML as a dead-end metabolite (data not shown). 4-ML itself as well as 4-methylmuconolactone methyl ester as reaction product after methylation were identified by GC-MS analyses (see Table 2).

### Intermediates of 2-ML degradation

**Fig 4:**
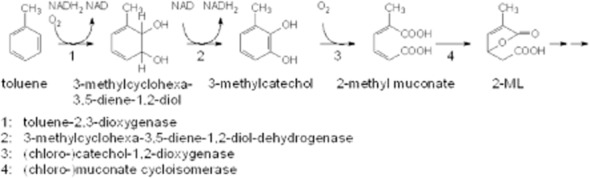
Postulated pathway of toluene degradation by strain *Burkholderia fungorum* FLU100 via 3–methylcyclohexa-3,5-diene-1,2-diol, 3-methylcatechol, 2-methylmuconic acid and 2-methyl-2-ene-lactone.

To identify further intermediates of 2-ML degradation pathway, analyses were performed by use of HPLCs with an evaporative light scattering detector (HPLC-ELSD) or a mass spectrometer (HPLC-MS). However, neither metabolites of 2-ML degradation were reproducibly detectable nor identification of detected compounds was possible. 2-methyl succinate as hypothetical intermediate of the 2-ML degradation pathway, however, was used as a substrate in conversion experiments. Whereas the reference substrate succinate was transformed without showing any lag phase, methyl substituted succinate was not converted at all by whole cells of FLU100 (see Supporting Information Table S5). Therefore, revelation of the complete degradation pathway downstream of the intermediate 2-ML was not successful. Maximum conversion rates of the identified intermediates of toluene degradation by FLU100 pre-grown on toluene are given in Table 1. The correlating degradation pathway is shown in Fig. 4.

## Concluding remarks

*Burkholderia fungorum* strain FLU100 was shown to mineralize toluene alone and simultaneously in any mixture with benzene and mono-halogen benzenes, even including fluorobenzene. The initial attack on all these substrates by a dioxygenase attacking the aromatic ring yielded into the corresponding 3-substituted cyclohexa-3,5-diene-1,2-diols, being metabolized via a dehydrogenase to the corresponding 3-(substituted) catechols. These catechols were nearly exclusively cleaved by a chlorocatechol-1,2-dioxygenase to the muconic acid derivatives. Based on a lack of *meta* cleavage activity, toluene is atypically cleaved by this *ortho* enzyme resulting in the formation of 2-methylmuconic acid, followed by 2-methyl-2-ene-lactone (2-ML) as the next steps. In contrast to literature, the latter intermediate is not a dead-end metabolite. The further degradation of 2-ML by so far unknown enzymes, induced by growth with toluene, remains yet to be elucidated.

## Experimental procedures

### Cultivation conditions of FLU100 (wild type and mutants)

Wild type of strain FLU100 was cultivated at 30°C in 500 ml glass flasks containing 200 ml of a mineral salt medium previously described (Dobslaw, 2003). About 20 μl of toluene, benzene or mono-halogen benzenes was directly added to this medium and flasks were incubated for 48–72 h. After adding 10–15 μL of substrate, cultures were further incubated overnight and harvested by centrifugation. The pellet was re-suspended in fresh medium.

The mutant P2R5 of strain FLU100 was obtained via transposon mutagenesis by mating the recipient FLU100 with the donor strain *E. coli* pCro2a (Onaca *et al*., 2007) and screening of nearly 16 800 mutants. The plasmid carries an ampicillin resistance gene for false-positive screening, whereas the Tn5 insert carries a kanamycin resistance gene and an *ori*-sequence. Mutants were screened by spreading cells on mineral medium plates containing 10 mmol l^−1^ glucose and 100 mg l^−1^ kanamycin. Colonies grown were transferred into liquid cultures (20 ml volume) with the same medium. The accumulation of intermediates of aromatic degradation was checked by adding 10 μl of toluene or another aromatic compound and 200 μl of a 1 M glucose solution after 48 h. Formation of intermediates was verified the next day via HPLC analyses.

For quantification of remaining concentrations of aromatics in the liquid medium via extraction and subsequent GC analyses, mixtures of aromatic compounds were supplied in DMFA with final concentrations of 2 to 3 mmol l^−1^ aromatics and 0.25% DMFA in the cultivation medium. Extraction was performed using dichloromethane.

In case of oxygen uptake experiments, the pellet was re-suspended in fresh mineral medium and culture was shaken at 30°C overnight without supplying substrates. As preparation, the culture was aerated, samples of 3 ml of suspension were filled into a 30°C tempered stirrer and oxygen uptake was recorded. For measurement of the substrate-specific oxygen uptake, 30 μL of a substrate solution (c = 0.1 mol L^−1^) was added.

In the event of crude extract experiments, the centrifuged pellets of strain FLU100, *P. knackmussii* B13 or *E. coli* Klon 4, were washed twice with saline solution and re-suspended in 15 ml of a Tris buffer solution (8 g l^−1^ Tris, pH 8.0). Cells were cracked by pressure release in two to three runs in a French pressure cell with 10 000 psi. The extract was centrifuged for 10 min at 14 000 rpm at 4°C and cell-free supernatant was stored on ice. At the conversion tests of catechols, 100 μL of crude extract was added to 1 ml of Tris buffer pH 8.0, and 900 μl of deionized water and 26 μl of ethylenediaminetetraacetic acid (c = 0.1 mol L^−1^) in a 4 ml fused silica cuvette. After aeration for 1 min, 20 μL of the tested catechol (c = 0.1 mol L^−1^) was added and the change in extinction at 260 nm was determined. In case of high turnover, the samples were measured by HPLC analyses at 210 nm and 260 nm wave lengths. Quantification of conversion rates (μmol l^−1^ min^−1^ mg protein^−1^) was performed via photometric measurement at maximum extinction wave lengths using extinction coefficients given by (Dorn and Knackmuss 1978). The concentration of protein was measured by the method of Bradford.

Conversion tests of catechols in whole cells of strain FLU100 were performed using cultures with optical densities of OD_546nm_ = 1.5–2.5. Cultures were firstly pre-grown on an aromatic substrate, centrifuged and re-suspended in new mineral medium. Relevant catechols were added in final concentrations of 3 mmol l^−1^. The concentrations of catechols and corresponding intermediates over time were observed by HPLC analyses.

2-ML as substrate for transformation tests was obtained via conversion of 3-methylcatechol by whole cells of strain FLU100 pre-grown on benzene. Directly after total transformation of 3-methylcatechol to 2-ML, reaction mixtures were centrifuged and the supernatant was directly used in subsequent conversion experiments as aqueous substrate solution. The solution can be stored at 4°C about 1 week. To avoid the induction of new enzymes during transformation of 2-ML, CAP as cytostatic drug was added (10 mg per 50 ml reaction volume). Actual concentrations of 2-ML in the reaction flasks were measured by HPLC analyses.

### Cultivation conditions of strains of E. coli

All strains of *E. coli* were pre-grown on lysogeny broth media containing 10 g of tryptone, 5 g of yeast extract and 5 g of sodium chloride per liter of deionized water. The medium was autoclaved at 121°C for 30 min. With the exception of strain pCro2a, 2.5 mmol l^–1^ of lactose was added. For cultivation of pCro2a, 100 mg l^−1^ of ampicillin and 50 mg l^−1^ of kanamycin were added. In case of *E. coli* Klon 4, 100 mg l^–1^ of kanamycin and in case of *E. coli* pST04, 100 mg l^−1^ of ampicillin and 10 mmol l^−1^ of glucose were added. Cultures were cultivated at 37°C overnight. Afterwards, the pellets of pST04 and Klon 4 were harvested and re-suspended in new LB-media with the corresponding additives and additional 0.25–0.4 mmol l^−1^ IPTG and 5–10 mmol l^−1^ of aromatic substrate (pST04) or 1 mmol l^−1^ of catechol (Klon 4). After 4 h, a significant blue colour appeared showing expression of the lac-regulated gene sequences. The production of substituted cyclohexa-3,5-diene-1,2-diols was directly measured per HPLC analyses at 260 nm. For production of muconic acids, Klon 4 was analogously prepared like FLU100 for crude extract measurements.

### Cultivation conditions of strains P. knackmussii B13

Strain B13 was cultivated at 30°C on a mineral medium containing benzoate or 3-chlorobenzoate as substrate. While a catechol-1,2-dioxygenase was induced in case of the former substrate, a chlorocatechol-1,2-dioxygenase was induced in the event of the latter substrate. The preparation was similar to that of crude extract measurements of FLU100.

## Supporting Information

**Fig. S1.** Influence of the pH value of the medium on the concentration of 2-methylmuconic acid as intermediate during conversion of 1.55 mmol toluene l^−1^ by strain FLU100 pre-grown on toluene. The chart for pH 5.0 fell out of the series because of reduced conversion rates of toluene as substrate. **Fig. S2.** Characterization and identification of formed ‘diendiol’ structures of benzene, toluene and fluorobenzene by mutant strain FLU100 P2R5 via HPLC analyses (column: ProntoSIL™ SC-04 Eurobond C18 column, 125 mm, 4 mm, i.d. 5 μm; solvent: H_2_O : CH_3_OH : H_3_PO_4_ (85 w/v%) = 74.9%: 25%: 0.1%). The flow rate was maintained at 1 ml min^−1^. **Table S1.** Conversion rates of aromatics in single, binary and ternary mixtures under different cultivation conditions. The corresponding generation times are given in the last column. B = benzene; CB = chlorobenzene; FB = fluorobenzene; T = toluene. **Table S2.** Specific oxygen uptake activities for the initial enzyme(s) of cells of FLU100 pre-grown on fluorobenzene, toluene or benzene. The value in parenthesis represents the number of independent batch cultures tested twice. The first value of each column describes the average value of oxygen uptake while the second one describes the variation. The absolute activity for fluorobenzene grown cells was 465 ± 77.2 units, 623.8 ± 243.7 units for toluene and 629.4 ± 190.1 units for benzene. One unit of oxygenase activity was defined as the conversion of 1 μg O_2_ l^−1^ min^−1^ OD^−1^. **Table S3.** Specific oxygen uptake activity of the (chloro)catechol-1,2-dioxygenase of strain FLU100 after growth on fluorobenzene (FB), toluene (T) or benzene (B). The value in parenthesis represents the number of independent batch cultures tested twice. The first value of each column describes the average oxygen uptake while the second one describes the variation. The absolute activity of fluorobenzene grown cells was 2090.5 ± 395.1 units, 189.4 ± 124.5 units for toluene and 88.7 ± 29.0 units for benzene. One unit of oxygenase activity was defined as the conversion of 1 μg O_2_ l^−1^ min^−1^ OD^−1^. **Table S4.** Identification of 3-methylcatechol (native form) and 2-methoxy-3-methylphenol (methylated form) as intermediate of toluene degradation of strain FLU100 by GC-MS analyses. The fragment with the highest intensity is normalized to 100% and other fragments are given as relative intensities. **Table S5.** Conversion rates (in mmol l^−1^ h^−1^ OD_546_^−1^) and generation times (in hours) of cells of FLU100 pre-grown on toluene or glucose as reference. n.d. = not detected or intensity not given in literature.
