# Supplementary material for: Degradation of toluene by ortho cleavage enzymes in Burkholderia fungorum FLU100
Source: Microb Biotechnol. 2014 Aug 18;8(1):143–54. doi: 10.1111/1751-7915.12147 (PMC4321380; doi:10.1111/1751-7915.12147)
Supplement: Supplementary file 1 — Fig. S1. Influence of the pH value of the medium on the concentration of 2-methylmuconic acid as intermediate during conversion of 1.55 mmol toluene l−1 by strain FLU100 pre-grown on toluene. The chart for pH 5.0 fell out of the series because of reduced conversion rates of toluene as substrate. Fig. S2. Characterization and identification of formed ‘diendiol’ structures of benzene, toluene and fluorobenzene by mutant strain FLU100 P2R5 via HPLC analyses (column: ProntoSIL™ SC-04 Eurobond C18 column, 125 mm, 4 mm, i.d. 5 μm; solvent: H2O : CH3OH : H3PO4 (85 w/v%) = 74.9%: 25%: 0.1%). The flow rate was maintained at 1 ml min−1. Table S1. Conversion rates of aromatics in single, binary and ternary mixtures under different cultivation conditions. The corresponding generation times are given in the last column. B = benzene; CB = chlorobenzene; FB = fluorobenzene; T = toluene. Table S2. Specific oxygen uptake activities for the initial enzyme(s) of cells of FLU100 pre-grown on fluorobenzene, toluene or benzene. The value in parenthesis represents the number of independent batch cultures tested twice. The first value of each column describes the average value of oxygen uptake while the second one describes the variation. The absolute activity for fluorobenzene grown cells was 465 ± 77.2 units, 623.8 ± 243.7 units for toluene and 629.4 ± 190.1 units for benzene. One unit of oxygenase activity was defined as the conversion of 1 μg O2 l−1 min−1 OD−1. Table S3. Specific oxygen uptake activity of the (chloro)catechol-1,2-dioxygenase of strain FLU100 after growth on fluorobenzene (FB), toluene (T) or benzene (B). The value in parenthesis represents the number of independent batch cultures tested twice. The first value of each column describes the average oxygen uptake while the second one describes the variation. The absolute activity of fluorobenzene grown cells was 2090.5 ± 395.1 units, 189.4 ± 124.5 units for toluene and 88.7 ± 29.0 units for benzene. One unit of oxygenase a [file mbt20008-0143-sd1.doc]

**Supplementary materials**

Table 1: Conversion rates of aromatics in single, binary and ternary mixtures under different cultivation conditions. The corresponding generation times are given in the last column.

B: benzene T: toluene FB: fluorobenzene CB: chlorobenzene

| Cultivation substrate / test substrate(s) | Substrate specific transformation rate [mg C/(L·h·OD)] | | | | Transformation rate total  [mg C/L·h·OD)] | Generation time g [h] |
| --- | --- | --- | --- | --- | --- | --- |
|  | B | T | FB | CB |  |  |
| B / B | 121.4 | - | - | - | 121.4 | 6.8 |
| B / T | - | 178.2 | - | - | 178.2 | 5.0 |
| B / FB | - | - | 81.2 | - | 81.2 | 14.2 |
| B / B + T | 56.2 | 63.2 | - | - | 119.4 | 6.6 |
| B / B + FB | 24.5 | - | 51.0 | - | 75.4 | 12.1 |
| B / B + FB + T | 24.9 | 41.8 | 20.2 | - | 86.9 | 8.7 |
| T / B | 53.4 | - | - | - | 53.4 | 8.3 |
| T / T | - | 89.6 | - | - | 89.6 | 4.8 |
| T / FB | - | - | 40.7 | - | 40.7 | 16.4 |
| T / CB | - | - | - | 51.4 | 51.4 | 6.4 |
| T / B + FB | 38.8 | - | 26.7 | - | 65.5 | 5.5 |
| T / B + T | 29.4 | 40.9 | - | - | 70.3 | 5.6 |
| T / FB + T | - | 41.1 | 32.7 | - | 73.8 | 8.5 |
| T / T + CB | - | 25.0 | - | 20.2 | 45.1 | 12.6 |
| T / B + FB + T | 25.0 | 41.5 | 21.1 | - | 87.5 | 8.9 |
| T / B + T + CB | 61.8 | 77.4 | - | 50.1 | 189.4 | 13.5 |
| FB / FB + T | - | 50.0 | 31.7 | - | 81.7 | 6.2 |
| FB / B + FB + T | 25.7 | 31.9 | 21.5 | - | 79.1 | 7.4 |

Table 2: Specific oxygen uptake activities for the initial enzyme(s) of cells of FLU100 pre-grown on fluorobenzene, toluene or benzene. The value in parenthesis represents the number of independent batch cultures tested twice. The first value describes the average value of oxygen uptake while the second one describes the variation. The absolute activity for fluorobenzene grown cells was 465 ± 77.2 units, 623.8 ± 243.7 units for toluene and 629.4 ± 190.1 units for benzene. One unit of oxygenase activity was defined as the conversion of 1 µg O_2_·L^-1^·min^-1^·OD^-1^.

|  | Specific activity fluorobenzene (7) [%] | Specific activity toluene (11) [%] | Specific activity benzene (7) [%] |
| --- | --- | --- | --- |
| **Mono-halogen aromatics** |  |  |  |
| fluorobenzene | 62.5 ± 11.2 | 24.3 ± 5.8 | 28.3 ± 10.6 |
| chlorobenzene | 89.9 ± 10.4 | 63.8 ± 12.2 | 125.4 ± 27.6 |
| bromobenzene | 92.7 ± 12.6 | 60.6 ± 6.1 | 119.0 ± 18.0 |
| 2-chlorotoluene | -1.5 ± 3.1 | -1.3 ± 2.2 | 2.9 ± 1.1 |
| **Methyl aromatics** |  |  |  |
| benzene | 59.2 ± 21.9 | 43.6 ± 9.6 | 42.8 ± 13.5 |
| toluene | 100.0 ± 4.2 | 100.0 ± 7.1 | 100.0 ± 6.6 |
| styrene | 27.0 ± 8.2 | 28.7 ± 8.4 | 43.8 ± 10.0 |
| cumene | 21.9 ± 10.6 | 15.8 ± 7.0 | 9.2 ± 5.7 |
| p-cymene | -3.7 ± 4.3 | -2.3 ± 3.0 | -4.0 ± 6.9 |
| o-xylene | -2.3 ± 2.9 | 0.2 ± 0.5 | -2.7 ± 3.5 |
| m-xylene | -1.4 ± 1.8 | -0.5 ± 1.3 | -0.3 ± 5.3 |
| p-xylene | 2.0 ± 1.8 | -0.9 ± 1.9 | 1.9 ± 0.4 |
| **Side chain oxidation** |  |  |  |
| benzyalcohol | 7.5 ± 2.7 | 7.3 ± 2.9 | 8.8 ± 4.5 |
| benzaldehyde | 105.0 ± 25.5 | 43.8 ± 11.2 | 34.2 ± 7.2 |
| benzoate | 1.3 ± 3.4 | -1.2 ± 2.9 | 5.6 ± 3.0 |
| o-toluate | 1.8 ± 2.4 | -2.8 ± 0.7 | -2.0 ± 1.5 |
| m-toluate | -0.1 ± 0.9 | 2.7 ± 2.8 | -2.0 ± 2.4 |
| p-toluate | 2.9 ± 1.9 | 4.0 ± 2.2 | -6.3 ± 6.8 |
| **Phenols** |  |  |  |
| phenol | 11.4 ± 3.5 | 16.0 ± 9.8 | 14.2 ± 3.4 |
| o-cresol | 5.5 ± 3.9 | 12.6 ± 5.5 | 10.2 ± 3.3 |
| m-cresol | 18.2 ± 4.5 | 19.9 ± 9.0 | 19.8 ± 5.9 |
| p-cresol | 6.2 ± 4.5 | 6.3 ± 5.9 | 12.3 ± 3.4 |
| 2-fluorophenol | 4.8 ± 2.4 | 5.1 ± 4.6 | 5.3 ± 1.1 |
| 3-fluorophenol | 13.6 ± 4.6 | 12.0 ± 3.3 | 10.8 ± 1.4 |
| 4-fluorophenol | 9.3 ± 4.1 | 18.7 ± 4.4 | 19.0 ± 5.9 |
| 2-chlorophenol | 0.8 ± 2.3 | 2.7 ± 2.4 | 3.1 ± 0.8 |
| 3-chlorophenol | 15.5 ± 4.3 | 16.8 ± 5.3 | 18.6 ± 6.9 |
| 4-chlorophenol | 14.1 ± 3.1 | 12.1 ± 4.5 | 20.1 ± 4.0 |
| **Others** |  |  |  |
| glucose | 12.8 ± 4.6 |  | -1.7 ± 0.8 |

Table 3: Specific oxygen uptake activity of the (chloro)catechol-1,2-dioxygenase of strain FLU100 after growth on fluorobenzene (FB), toluene (T) or benzene (B). The value in parenthesis represents the number of independent batch cultures tested twice. The first value describes the average oxygen uptake while the second one describes the variation. The absolute activity of fluorobenzene grown cells was 2090.5 ± 395.1 units, 189.4 ± 124.5 units for toluene and 88.7 ± 29.0 units for benzene. One unit of oxygenase activity was defined as the conversion of 1 µg O_2_·L^-1^·min^-1^·OD^-1^.

B: benzene T: toluene FB: fluorobenzene

|  | Specific activity FB with reference T (7) | Specific activity FB with reference catechol (7) | Specific activity T with reference T (7) | Specific activity T with reference catechol (7) | Specific activity B with reference T (7) | Specific activity B with reference catechol (7) |
| --- | --- | --- | --- | --- | --- | --- |
| **Catechols** |  |  |  |  |  |  |
| catechol | 465.7 ± 128.0 | 100.0 ± 5.1 | 30.5 ± 10.5 | 100.0 ± 9.6 | 16.0 ± 5.5 | 100 ± 12.3 |
| 3-methylcatechol | 598.6 ± 128.6 | 136.1 ± 16.3 | 43.2 ± 14.6 | 145.2 ± 32.9 | 80.5 ± 25.4 | 571.6 ± 247.2 |
| 4-methylcatechol | 504.8 ± 87.9 | 119.8 ± 25.6 | 20.9 ± 13.4 | 68.1 ± 34.2 | 33.8 ± 8.2 | 232.9 ± 91.1 |
| 3-methoxycatechol | 400.4 ± 34.5 | 108.7 ± 29.7 | 17.8 ± 9.3 | 50.0 ± 23.2 | 13.5 ± 3.7 | 91.2 ± 36.4 |
| 3-fluorocatechol | 123.4 ± 11.5 | 33.3 ± 8.6 | 4.2 ± 5.5 | 8.4 ± 13.8 | 1.5 ± 3.4 | 8.6 ± 21.4 |
| 4-fluorocatechol | 376.4 ± 99.7 | 83.8 ± 4.3 | 28.1 ± 7.0 | 97.0 ± 43.9 | 31.8 ± 12.7 | 195.5 ± 51.1 |
| 3-chlorocatechol | 275.8 ± 32.0 | 65.9 ± 18.3 | 19.7 ± 10.2 | 63.7 ± 20.5 | 33.5 ± 9.7 | 235.5 ± 83.1 |
| 4-chlorocatechol | 95.1 ± 11.2 | 23.9 ± 10.2 | 13.0 ± 5.6 | 43.2 ± 20.1 | 23.4 ± 9.9 | 151.0 ± 72.9 |
| 2,3-dihydroxybenzoate | -3.3 ± 0.7 | -0.9 ± 0.2 | -0.7 ± 1.9 | -2.7 ± 6.5 | -1.6 ± 1.5 | -7.6 ± 6.8 |
| 3,4-dihydroxybenzoate | 9.4 ± 2.3 | 3.1 ± 1.4 | 11.7 ± 6.3 | 38.0 ± 22.4 | 16.4 ± 5.4 | 125.8 ± 74.7 |

Table 4: Identification of 3-methylcatechol (native form) and 2-methoxy-3-methylphenol (methylated form) as intermediate of toluene degradation of strain FLU100 by GC-MS analyses. The fragment with the highest intensity is normalised to 100 % and other fragments are given as relative intensities.

| Identity | Fragment [m/z] | Intensity [%] | Description |
| --- | --- | --- | --- |
| 3-methylcatechol | 124  123  105  95  78  77 | 100.0  35.0  7.4  4.0  24.0  11.2 | M^+^  M^+^- H  M^+^- H - H_2_O  M^+^- HCO  M^+^ - HCO - OH  M^+^- CH_3_– OH – OH - H |
| 2-methoxy-3-methylphenol | 138  123  95  77 | 100.0  94.2  6.8  12.7 | M^+^  M^+^ - CH_3_  M^+^ - CH_3_ - HCO  M^+^ - OCH_3_ – OH – CH |

Table 5: Conversion rates (in mmol·L^-1^·h^-1^·OD_546_^-1^) and generation times (in h) of cells of FLU100 pre-grown on toluene or glucose as reference.

-: not analysed n.d. not detectable

|  | Toluene | | | Glucose | | |
| --- | --- | --- | --- | --- | --- | --- |
|  | without CAP | with CAP | generation time [h] | without CAP | with CAP | Generation time [h] |
| 3-methylcatechol | 0.18 | - | 8.6 | - | - | - |
| acetate | 0.80 | n. d. | 9.5 | 0.69 | n. d. | 19.1 |
| pyruvate | 1.48 | n. d. | 10.1 | - | - | - |
| succinate | 3.33 | n. d. | 2.7 | - | - | - |
| methyl succinate | n. d. | n. d. | 30.9 | - | - | - |
| adipate | 0.62 | 0.01 | 4.5 | - | - | - |
| β-hydroxy butyrate | 0.79 | n. d. | 7.3 | 0.47 | 0.11 | 13.6 |
| γ-hydroxy butyrate | n. d. | n. d. | 51.2 | 0.03 | 0.03 | > 100 |
| 2-methyl butyrate | n. d. | n. d. | > 100 | n. d. | n. d. | 98.6 |
| γ-hydroxy butyrolactone | n. d. | 0.06 | > 100 | n. d. | 0.01 | > 100 |
| malonate | n. d. | n. d. | > 100 | n. d. | n. d. | > 100 |
| 3-oxoglutarate | 0.37 | 0.48 | 10.5 | 0.18 | 0.04 | 5.1 |
| 2-ML | 0.41 | 0.04 | 5.4 | - | - | - |


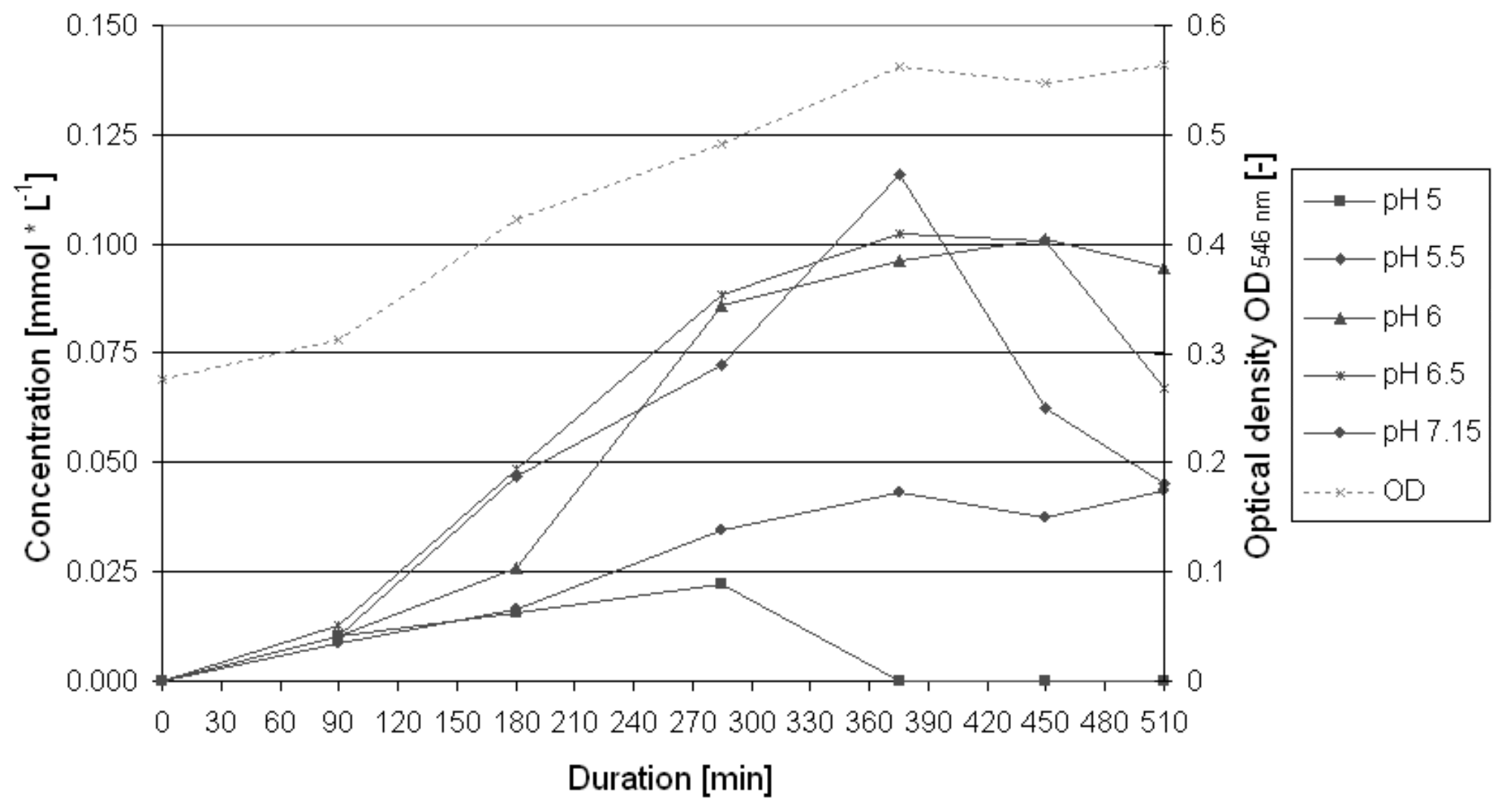


Fig 1: Influence of the pH value of the medium on the concentration of 2-methylmuconic acid as intermediate during conversion of 1.55 mmol toluene·L^-1^ by strain FLU100 pre-grown on toluene. The chart for pH 5.0 fell out of the series due to reduced conversion rates of toluene as substrate.


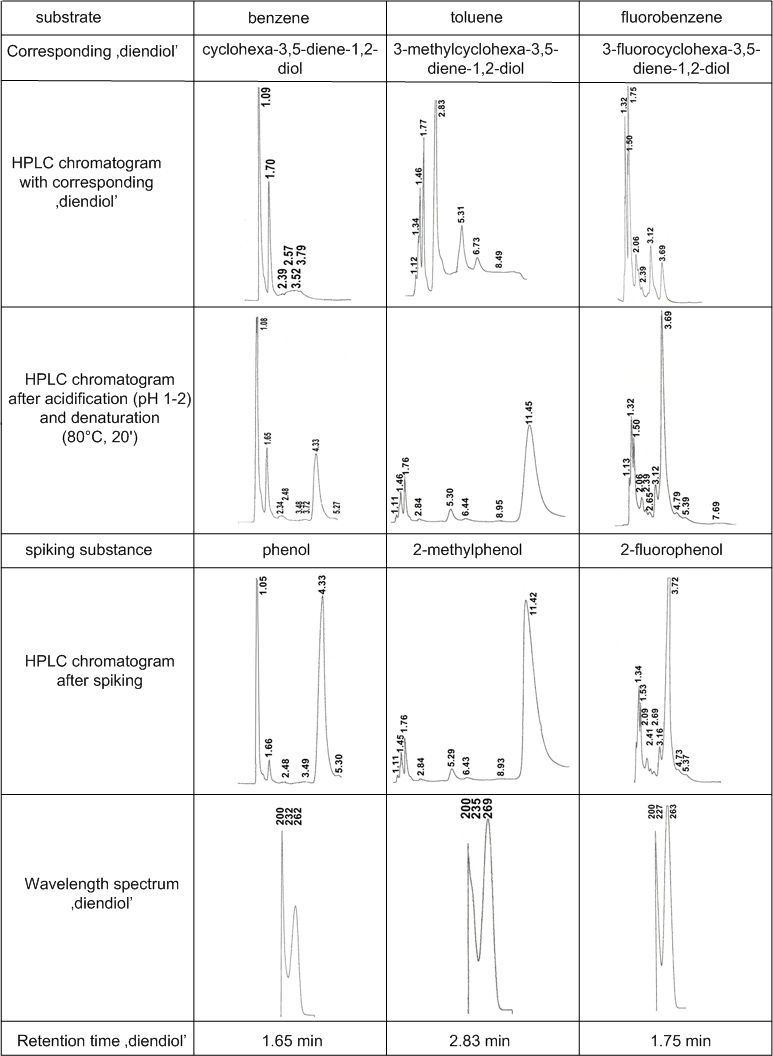


Fig 2: Charakterisation and identification of formed ‘diendiol’ structures of benzene, toluene and fluorobenzene by mutant strain FLU100 P2R5 via HPLC analyses (column: ProntoSIL™ SC-04 Eurobond C18 column, 125 mm · 4 mm, i.d. 5 µm; solvent: H_2_O : CH_3_OH : H_3_PO_4_ (85 w/v%) = 74.9 % : 25 % : 0.1 %). The flow rate was maintained at 1 mL · min^-1^.
